# Quantifying technical load and physical activity in professional soccer players during pre-season matches with IMU technology

**DOI:** 10.3389/fphys.2023.1274171

**Published:** 2023-11-30

**Authors:** José Augusto Losada-Benitez, Francisco Javier Nuñez-Sánchez, José Carlos Barbero-Álvarez

**Affiliations:** ^1^ Head of Performance, FC Persepolis, Tehran, Iran; ^2^ Physical Performance and Sports Research, Pablo de Olavide University, Sevilla, Spain; ^3^ Faculty of Education and Sport Science, University of Granada, Melilla, Spain

**Keywords:** soccer (football), monitoring, technical load, physical load, quantifying

## Abstract

This study aimed to record, analyze and quantify professional soccer players’ technical (TL) and physical load (PL) in friendly matches to compare their records during the first and second halves and between players with different positions. Eighteen professional soccer players, 24.6 ± 2.7 years, 1.78 ± 0.3 height (m), 74.6 ± 4.5 body mass (kg), 9.8 ± 2.2 body fat (%), and 65.6 ± 2.7 maximal oxygen consumption (VO_2max_, ml·kg^-1^·min^-1^) were monitored during six preseason friendly matches to analyze the activity profile using technical and physical variables through inertial measurement unit (IMU). No significant differences were found between the periods for the TL and PL. Significant differences were found between specific positions: Full Back (FB: n = 4), Central Defender (CD: n = 3), Midfielder (MD: n = 4), Winger (WG: n = 4), and Forward (FW: n = 3), both the TL and PL. We conclude that the PL profile based on his playing position is independent of the development of the PL shown during friendly matches. The monitoring, quantifying, and controlling of the TL added to the PL provides a more holistic vision of soccer players in friendly matches. The relative ease IMU application technology offers an alternative with less time-cost and more significant benefits than other types of technologies applied up to now.

## Introduction

Soccer performance is multifactorial and requires training programs that combine technical, tactical, and psychological aspects (Stølen et al., 2005). That´s why the more incredible the information and control of these types of variables, the more informed decision-making by the members of the coaching staff, the greater the chances of improving the performance of soccer players and increasing the chances of success (Buchheit et al., 2014; Taberner et al., 2020). In matches, players typically transition between short, high-intensity efforts and long periods of low-intensity activities (Bangsbo et al., 2006). However, performance in soccer depends on these more physiological factors. Still, there are various factors, possibly more determinants, such as technical, tactical, or mental, which also greatly influence performance (Torreño, 2017). Currently, the design of soccer training sessions and tasks are based on technical-tactical and physical actions directly related to simulated game situations, to the detriment of analytical studies, due to the close relationship of these actions with the activity carried out in the matches (Carling, 2013; Barrett et al., 2020). However, the design and implementation of these training tasks can cause different results after their completion since there are endless variables that directly influence the development obtained, such as the dimension of the pitch, the number of participating players, the number of touches allowed, the tactical instructions to comply with or the work-rest ratios between series-repetitions (Akenhead and Nassis, 2016). In recent years, the means for monitoring and quantifying PL have proliferated, both in training sessions and matches using GPS technology (Barbero-Álvarez et al., 2009; Akenhead et al., 2013; Malone et al., 2015; Suarez-Arrones et al., 2015; Akenhead and Nassis, 2016; Barrett et al., 2020). However, this physical analysis does not demand special attention to the technical-tactical demands to which soccer players are subjected (Barnes et al., 2014) and is called TL. Until recently, TL data was obtained through complex infrastructures, such as video analysis and semi-automatic recording systems (Di Salvo et al., 2006; Castellano et al., 2014; Arjol-Serrano et al., 2021) or local positioning systems (Curtis et al., 2019). Recently, inertial measurement devices (IMU) have been designed (Edwards et al., 2019), which, placed in the soccer player´s boot, can represent a low-cost option that improves the task of monitoring the soccer player´s TL and PL. Some studies have shown the ability to monitor both TL and PL in English professional teams over an extended period of the season, analyzing the tasks of training sessions with male and female players (Barrett et al., 2020; Marris et al., 2021; Lewis et al., 2022; Myhill et al., 2022). Previous studies on monitoring TL and PL in soccer players have primarily focused on official matches or without considering TL (Barnes et al., 2014; Suarez-Arrones et al., 2015; Akenhead and Nassis, 2016), this is where we find a gap in information and knowledge, which P.S. Bradley (2020) himself questions, running from a “traditional” to an “integrated” approach to understanding the demands of the game. Therefore, there needs to be more research in understanding TL and PL profiles, specifically during preseason and friendly. The objectives are to record, analyzing and quantify the TL and PL of professional soccer players in friendly matches, compare their records during the first and second halves, and professional soccer players during friendly matches. Specifically, the study aims to compare TL and PL records between the first and second halves of and among players in different specific positions in the preseason. We hypothesize that there could be significant differences in TL and PL between the first and second halves of matches. Additionally, we expect to observe variations in TL and PL among players with different specific positions.

## Material and methods

### Participants

A total of 18 professional soccer players (n = 18) aged 24.6 ± 2.7 years, 1.78 ± 0.32 height (m), 74.6 ± 4.5 body mass (kg), 23.54 ± 2.7 body mass index (kg.m^2^), 9.8% ± 2.2% body fat, and 65.6 ± 2.7 maximal oxygen consumption (VO_2max_, ml·kg^-1^·min^-1^) belonging to the same team, took part in this study. The inclusion criteria were only data from outfield players who participated a minimum of 45 min in one of the two periods. In addition, participants were required to be in good health and free from any injuries that could affect their performance during the matches. At the same time, the exclusion criteria were one friendly match due to their extension of more than 90 min. Also, goalkeepers were excluded from the study to focus specifically on outfield players and their activity profiles. In the study, 103 records were made, 59 for the first period and 44 for the second one. The players were classified according to their specific position: Full Back (FB: n = 4), Central Defender (CD: n = 3), Midfielder (MD: n = 4), Winger (WG: n = 4), and Forward (FW: n = 3). All of them were previously informed about the object of study and provided their signed informed consent, following the indications of the Declaration of Helsinki (2013). Before starting the study, it was approved by the ethics committee of Pablo de Olavide University with code 0398-N17.

### Sample size

To determine the sample size for this study, we employed G*Power software 3.1.9.7 (Faul et al., 2007) and conducted *a priori* calculations using the t-test family. We set the significance level (α) to 0.05, the desired power (1 - β error probability) to 0.80, and based on the effect size on previous studies (Nobari et al., 2021a; Nobari et al., 2021b), ranging from medium to high. The analysis indicated that a total sample size of 16 participants would yield an actual power of 81% for the present analysis.

### Study design

The study employed a descriptive design, observing the methodology applied in data collection. Data were collected during friendly matches in the 2020-21 preseason (August-October) involving a professional soccer team competing in the third tier of Spanish soccer. The preseason period is characterized by a high PL for the players (Ade et al., 2016). Furthermore, the distribution of playing minutes in these friendly matches was evenly spread across all members of the team’s squad. Throughout the 7-week preparatory period, the team conducted 38 training sessions and participated in 7 friendly matches (with one match excluded from this study). The matches were played 5–7 days apart in the morning, between 10 and 11 a.m., all of them belonging to the same competitive level as us. All anthropometric measurements, body composition and VO_2max_ were performed before the preseason.

### Process and variables

#### Anthropometric, body composition and maximal oxygen consumption (VO_2max_)

The anthropometric, body composition and maximal oxygen consumption (VO_2max_) measurements were performed by specialist at the Andalusian Sport Medicine Center (https://www.juntadeandalucia.es/organismos/turismoculturaydeporte/areas/deporte/medicina-deportiva/sedes-camd/paginas/camd-cadiz.html) during 24th to 26th August 2020. Laboratory testing was conducted between 9 a.m. and 12 p.m. with ambient temperature between 22°C and 24°C. To measure height and body mass, the participants stood without shoes and with only shorts. For both measurements, a portable stadiometer (accuracy of ±5 mm) and balance weighting scales (accuracy of ±0.1 kg) (Seca model 207, Germany) were used.

Body mass index (kg/m^2^) was calculated using body mass/height^2^. Measuring the skinfold thickness at seven sites (chest, axilla, triceps, abdominal, subscapular, suprailiac and thigh) using a calliper (Holtain Skinfold Caliper, Holtain, UK). One experienced anthropometrist carried out all the anthropometric tests following the anthropometric measurement protocols established by the International Society for the Advancement of Kinanthropometry (ISAK). The percentage of body fat was calculated following Faulkner (1966).

A maximal exercise test on a treadmill (TM Trackmaster, United States) with a continuous and incremental protocol was made to calculate VO_2max_ designed by the components of the sports medicine service (Andalusian Sport Medicine Center). The initial speed was 9 km/h^-1^ for 3 min, then increased by 1 km/h every minute until exhaustion occurred within 10–15 min for all subjects. Maximal oxygen uptake was measured during both tests via a breath-by-breath gas analyzing system (Quark b2, Cosmed Co., Rome, Italy). The VO_2max_ with the highest VO_2_ was calculated when a plateau in O_2_ consumption was reported despite an increased workload. All these measures are considered as dependent variables in this study.

#### Monitoring technical load (TL) and physical load (PL)

**FIGURE 1:**
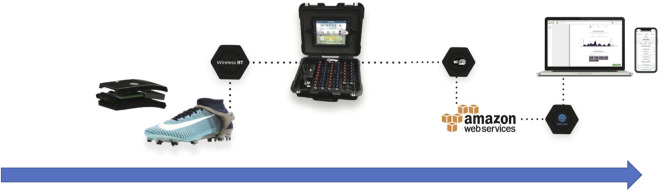
Timeline of the collecting data process using IMU technology (Playermaker™).

The players’ demands during friendly matches were monitored using an IMU technology-based data collection instrument (Figure 1). Smart motion devices (Playermaker™, Tel Aviv, Israel) were directly mounted on the soccer players’ boots to quantify TL and PL. Each IMU device incorporated two components from the MPU-9150 multi-chip (InvenSense, California, United States), which included a 16 g triaxial accelerometer and a 2000°/sec-1 triaxial gyroscope. Previous studies have demonstrated the excellent inter-unit reliability of these devices for all PL variables compared to GPS devices (Waldron et al., 2021). Similarly, when comparing TL variables with video analysis, these units have shown validity and reliability (Marris et al. (2021). Prior to the start of each match, following the completion of warm-up activities, each player was provided with an IMU device inserted into a silicone flange, which was placed beneath the lateral malleolus of their foot. Subsequently, after each match, the devices were placed in a docking box connected via Bluetooth to an iPad (Apple Inc., California). In this setup, each device downloaded the recorded data into Playermaker™ Dashboard software (v.3.22.0.02) for subsequent processing and analysis.

The TL variables analyzed have been: Total Touches (TT: number (#) of times the ball hits the player’s feet); Releases (REL: number (#) of times the player throws the ball with his foot); Total Possessions (TP: number (#) of times the player maintains possession of the ball); One touch (1T: number (#) of times the player contacts the ball without reception); Short Possessions (SP: number (#) of times the player maintains possession of the ball for no more than 2.5 s); Long Possessions (LP: number (#) of times the player retains control of the ball for more than 2.5 s); Receptions (RC: number (#) of times the player receives the ball); Release Velocity (RV: speed quantified in meters per second (m/s), with which the player performs the mechanical gesture at the moment of hitting the ball); Release Index (RI: is an indicator that combines the volume and intensity of each hit by the player and is presented as a single value (Arbitrary Units, AU, numeric) (Lewis et al., 2022).

The PL variables analyzed have been: Top Speed ​​(TS: highest speed peak (m/s) reached by a player); Distance Covered (DC: amount of total distance (m) traveled in meters); Work Rate (WR: indicator (m/min) of load measured in amount of distance traveled in meters (m) between the time measured in minutes (min); High Intensity Distance Covered (HIDC: total amount of distance (m) covered in a speed range greater than 4.1 m/s; Sprint Distance Covered (SDC: total distance (m) traveled in a range greater than speed of 5.83 m/s); Number of Sprints (SP: number (#) of times the player reaches a speed greater than 5.83 m/s); Distance Traveled Zone 1 (DTZ1: total amount of distance (m) traveled in meters in a speed range of 0.0–2.5 m/s); Distance Traveled Zone 2 (DTZ2: Total amount of distance (m) traveled in meters in a speed range of 2.5–4.17 m/s); Distance Traveled Zone 3 (DTZ3: total amount of distance (m) traveled in meters in a speed range of 4.17–5.0 m/s); Zone 4 Distance Traveled Zone 4 (DTZ4: total amount of distance (m) traveled in meters in a speed range of 5.0–5.83 m/s), Distance Traveled Zone 5 (DTZ5: amount of total distance (m) traveled in meters in a speed range of 5.83–6.66 m/s); Distance Traveled Zone 6 (DTZ6: amount of total distance (m) traveled in meters in a speed range greater than 6.66 m/s); Acceleration/Deceleration Actions (ADA: number (#) of times the player performs an intense change of direction and speed variation, in an accelerated or decelerated manner, in a speed range greater than 2.6 m/s^2^).

The data corresponding to TL variables: TT, LAN, REL, 1T, SP, LP, RC and RV and, to PL variables: HIDC, SDC, SP and ADA, are presented both in absolute values and relative to the time of game. For this, the data obtained in the first half’s friendly matches (average and standard deviation) are differentiated from those of the second period.

#### Statistical analysis

All variables were presented as mean values and standard deviations. The normal distribution of the data sets was assessed using the Shapiro-Wilk normality test. To analyze the differences between the first and second halves of technical load (TL) and physical load (PL), paired sample t-tests were performed. Additionally, a one-way analysis of variance (ANOVA) was utilized to compare the mentioned variables among playing positions during the preseason and a Bonferroni *post hoc* test was conducted to further investigate significant differences. A significance level of 95% was employed to determine statistical significance.

The effect size (ES) for the difference between variables was evaluated using Cohen’s d (Cohen, 1998). A value of d < 0.1, 0.1 to 0.20, 0.20 to 0.50, 0.50 to 0.80, and >0.80 was considered trivial or no effect, small, moderate, large, and very large, respectively. The SPSS software for Windows (v. 26; IBM, Chicago, United States) was used for data analysis.

## Results

The results obtained for the variables TL and PL are shown in Tables 1, 2, respectively. When comparing the TL for the first and second periods, no significant differences were observed in any of the variables analyzed. In the analysis of the PL variables, we found that WR (m/min) (p = ≤ 0.001, ES: 0.92), DC (m) (*p* = 0.02, ES: 0.57), HIDC (m) (*p* = 0.01, ES: 0.27), HIDC (m/min) (p = ≤ 0.001, ES: 0.36), SDC (m) (*p* = 0.02, ES: 0.21), SDC (m/min) (*p* = 0.01, ES: 0.23), SP (#) (*p* = 0.04, ES: 0.29), SP (#/min) (*p* = 0.01, ES: 0.37), DTZ3 (*p* = 0.01, ES: 0.31), DTZ4 (*p* = 0.01, ES: 0.26) and DTZ5 (*p* = 0.02, ES: 0.24), generate significantly higher values in the first part than in the second.

**TABLE 1:** Descriptive analysis of TL variables in absolutes (#) and relatives (#/min) values concerning time and speed releases (m/s).

TL variables	1st half	2nd half	t-test (p)	ES (d)	%Dif
TT (#)	47.0 ± 18.5	48.7 ± 26.4	0.57	−0.08	−3.62
TT (#/min)	1.03 ± 0.40	1.03 ± 0.51	0.92	0.00	0.00
REL (#)	16.4 ± 9.6	15.6 ± 10.7	0.77	0.12	7.15
REL (#/min)	0.36 ± 0.21	0.33 ± 0.23	0.55	0.18	11.11
TP (#)	17.7 ± 9.4	19.3 ± 10.4	0.47	−0.13	−7.08
TP (#/min)	0.39 ± 0.20	0.41 ± 0.23	0.68	−0.05	−2.56
1T (#)	6.0 ± 3.7	6.5 ± 3.2	0.36	−0.16	−9.24
1T (#/min)	0.13 ± 0.08	0.14 ± 0.07	0.55	−0.13	−7.69
SP (#)	4.8 ± 3.7	5.1 ± 3.9	0.48	−0.06	−4.97
SP (#/min)	0.11 ± 0.08	0.11 ± 0.08	0.66	0.00	0.00
LP (#)	6.9 ± 4.3	7.4 ± 5.1	0.44	−0.11	−7.12
LP (#/min)	0.15 ± 0.09	0.16 ± 0.11	0.66	−0.10	−6.67
RC (#)	11.7 ± 6.7	12.4 ± 8.1	0.41	−0.10	−6.23
RC (#/min)	0.26 ± 0.15	0.27 ± 0.17	0.15	−0.06	−3.85
RV Avg (m/s)	13.6 ± 1.1	13.7 ± 1.4	0.90	−0.11	−1.03
RV Max (m/s)	19.0 ± 1.5	19.3 ± 1.5	0.90	−0.20	−1.53
RI	22.6 ± 13.0	21.1 ± 14.6	0.96	0.08	4.82
RI/min	0.48 ± 0.29	0.46 ± 0.33	0.72	0.07	4.17

**Note:** TT: total touches; REL: releases; TP: total possessions; 1T: one touch; SP: short possession; LP: long; RC: receptions; RV: release velocity; RI: Release Index. Data are shown as mean ± standard deviation.

**TABLE 2:** Descriptive analysis of PL variables in absolutes (#; m) and relatives (#/min; m/min) values, concerning time.

PL variables	1st half	2nd half	t-test (p)	ES (d)	%Diff
TS (m/s)	7.4 ± 0.5	7.5 ± 0.4	0.65	−0.20	−1.22
DC (m)	4968.2 ± 477.0	4600.7 ± 828.2	0.02*	0.57	7.40
WR (m/min)	108.6 ± 10.5	96.5 ± 16.2	≤0.001*	0.92	11.16
HIDC (m)	1255.4 ± 967.1	1027.8 ± 651.8	0.01*	0.27	18.13
HIDC (m/min)	27.4 ± 21.0	21.7 ± 14.4	≤0.001*	0.36	23.91
SDC (m)	298.0 ± 364.6	232.0 ± 229.2	0.02*	0.21	22.14
SDC (m/min)	6.5 ± 7.9	4.9 ± 5.0	0.01*	0.23	24.46
SP (#)	20.3 ± 9.8	17.7 ± 7.3	0.04*	0.29	12.68
SP (#/min)	0.44 ± 0.22	0.37 ± 0.15	0.01*	0.37	15.91
DTZ1 (m)	2106.6 ± 436.0	2107.6 ± 513.0	0.24	0.00	−0.03
DTZ2 (m)	1606.8 ± 592.6	1464.6 ± 586.3	0.52	0.24	8.85
DTZ3 (m)	573.5 ± 299.5	490.6 ± 229.4	0.01*	0.31	14.44
DTZ4 (m)	383.9 ± 347.4	305.2 ± 239.0	0.01*	0.26	20.52
DTZ5 (m)	222.5 ± 282.5	165.5 ± 167.1	0.02*	0.24	25.62
DTZ6 (m)	75.5 ± 93.7	66.5 ± 73.0	0.13	0.11	11.87
ADA (#)	27.6 ± 7.6	25.2 ± 9.8	0.24	0.28	8.66
ADA (#/min)	0.60 ± 0.16	0.53 ± 0.20	0.07	0.40	11.67

**Note:** TS: top speed; DC: distance covered; WR: work rate; HIDC: high intensity distance covered; SDC: sprint distance covered; SP: number of sprints; DTZ1: Distance Traveled Zone 1; DTZ2: Distance Traveled Zone 2; DTZ3: Distance Traveled Zone 3; DTZ4: Distance Traveled Zone 4; DTZ5: Distance Traveled Zone 5; DTZ6: Distance Traveled Zone 6; ADA: Acceleration/Deceleration Actions. Data are shown as mean ± standard deviation. * Significant differences between the first and second half.

Tables 3, 4 show the results for each TL and PL variable, respectively, attending to the different positions for players who completed the first half. Significant differences by position are observed for both TL variables and PL variables. Considering TL variables by specific positions, the MDs are the ones that generated a more excellent record of TT without reaching significant differences with CD (*p* = 0.84) but obtaining significant differences with FB (*p* = 0.01) and with FW (*p* ≤ 0.001).

**TABLE 3:** Descriptive analysis of TL variables by playing positions.

TL variables	FB (4 players)	CD (3 players)	MD (4 players)	WG (4 players)	FW (3 players)
TT (#)	43.48 ± 15.99^b^ ^,^ ^c^ ^,^ ^d^ ^,^ ^e^	54.86 ± 22.47^a^ ^,^ ^e^	56.29 ± 18.33^a^ ^,^ ^e^	49.17 ± 15.82^a^ ^,^ ^e^	30.50 ± 13.17^a^ ^,^ ^b^ ^,^ ^c^ ^,^ ^e^
TT (#/min)	0.95 ± 0.34^c^ ^,^ ^d^ ^,^ ^e^	1.18 ± 0.50^e^	1.23 ± 0.40^a^ ^,^ ^e^	1.26 ± 1.50^a^ ^,^ ^e^	0.66 ± 0.27^a^ ^,^ ^b^ ^,^ ^c^ ^,^ ^e^
REL (#)	15.87 ± 7.36^c^ ^,^ ^e^	22.10 ± 12.76^d^ ^,^ ^e^	20.93 ± 9.01^a^ ^,^ ^d^ ^,^ ^e^	12.75 ± 6.05^b^ ^,^ ^c^	7.20 ± 6.23^a^ ^,^ ^b^ ^,^ ^c^
REL (#/min)	0.34 ± 0.16^c^ ^,^ ^e^	0.48 ± 0.28^d^ ^,^ ^e^	0.46 ± 0.19^a^ ^,^ ^d^ ^,^ ^e^	0.28 ± 0.12^b^ ^,^ ^c^	0.15 ± 0.13^a^ ^,^ ^b^ ^,^ ^c^
TP (#)	18.65 ± 8.03^b^ ^,^ ^e^	24.62 ± 11.51^d^ ^,^ ^e^	21.86 ± 8.96^d^ ^,^ ^e^	15.42 ± 6.27^b^ ^,^ ^c^ ^,^ ^e^	8.00 ± 6.60^a^ ^,^ ^b^ ^,^ ^c^ ^,^ ^d^
TP (#/min)	0.41 ± 0.17^e^	0.52 ± 0.25^d^ ^,^ ^e^	0.48 ± 0.19^d^ ^,^ ^e^	0.33 ± 0.13^b^ ^,^ ^c^ ^,^ ^e^	0.17 ± 0.14^a^ ^,^ ^b^ ^,^ ^c^ ^,^ ^d^
1T (#)	7.09 ± 3.30^e^	7.05 ± 3.34^e^	7.64 ± 3.79^d^ ^,^ ^e^	5.33 ± 3.45^c^	2.40 ± 2.07^a^ ^,^ ^b^ ^,^ ^c^
1T (#/min)	7.09 ± 3.30^e^	7.05 ± 3.34^e^	7.64 ± 3.79^d^ ^,^ ^e^	5.33 ± 3.45^c^	2.40 ± 2.07^a^ ^,^ ^b^ ^,^ ^c^
SP (#)	4.70 ± 3.42^b^ ^,^ ^e^	7.24 ± 4.01^a^ ^,^ ^d^ ^,^ ^e^	6.07 ± 3.20^d^ ^,^ ^e^	3.00 ± 2.66^b^ ^,^ ^c^	1.70 ± 2.06^a^ ^,^ ^b^ ^,^ ^c^
SP (#/min)	0.10 ± 0.07^b^ ^,^ ^e^	0.16 ± 0.09^a^ ^,^ ^d^ ^,^ ^e^	0.13 ± 0.07^d^ ^,^ ^e^	0.06 ± 0.06^b^ ^,^ ^c^	0.04 ± 0.04^a^ ^,^ ^b^ ^,^ ^c^
LP (#)	6.09 ± 3.36^b^ ^,^ ^d^	9.57 ± 5.83^a^ ^,^ ^e^	8.14 ± 4.66^e^	7.08 ± 2.68^a^	2.90 ± 3.81^b^ ^,^ ^c^
LP (#/min)	0.13 ± 0.07^b^ ^,^ ^d^ ^,^ ^e^	0.21 ± 0.13^a^ ^,^ ^e^	0.18 ± 0.10^e^	0.15 ± 0.06^a^	0.08 ± 0.08^a^ ^,^ ^b^ ^,^ ^c^
RC (#)	10.78 ± 4.80^b^ ^,^ ^c^ ^,^ ^e^	16.81 ± 9.31^a^ ^,^ ^d^ ^,^ ^e^	14.21 ± 5.83^a^ ^,^ ^d^ ^,^ ^e^	10.08 ± 4.21^b^ ^,^ ^c^	5.60 ± 5.15^a^ ^,^ ^b^ ^,^ ^c^
RC (#/min)	0.24 ± 0.10^c^ ^,^ ^e^	0.70 ± 1.54^d^ ^,^ ^e^	0.31 ± 0.12^a^ ^,^ ^d^ ^,^ ^e^	0.22 ± 0.09^b^ ^,^ ^c^	0.12 ± 0.11^a^ ^,^ ^b^ ^,^ ^e^
RV Avg (m/s)	13.63 ± 0.77^bi^	14.47 ± 0.62^a^ ^,^ ^c^ ^,^ ^e^	13.48 ± 0.91^b^	13.86 ± 1.08^b^	13.05 ± 1.00^b^
RV Max (m/s)	19.59 ± 1.34^c^	20.09 ± 1.96^c^	18.55 ± 1.03^a^ ^,^ ^b^	19.01 ± 2.05	17.83 ± 1.06
RI	21.34 ± 9.26^b^ ^,^ ^c^ ^,^ ^e^	32.01 ± 18.61^a^ ^,^ ^d^ ^,^ ^e^	28.04 ± 11.71^a^ ^,^ ^d^ ^,^ ^e^	17.49 ± 7.77^b^ ^,^ ^c^ ^,^ ^e^	9.22 ± 7.85^a^ ^,^ ^b^ ^,^ ^c^ ^,^ ^d^
RI/min	0.47 ± 0.21^b^ ^,^ ^c^ ^,^ ^e^	0.71 ± 0.41^a^ ^,^ ^d^ ^,^ ^e^	0.62 ± 0.26^a^ ^,^ ^d^ ^,^ ^e^	0.39 ± 0.17^b^ ^,^ ^c^ ^,^ ^e^	0.20 ± 0.17^a^ ^,^ ^b^ ^,^ ^c^ ^,^ ^d^

^a^ (significant differences from FB).

^b^ (significant differences from CD).

^c^ (significant differences from MD).

^d^ (significant differences from WG).

^e^ (significant differences from FW). TT: total touches; REL: releases; TP: total possessions; 1T: one touch; SP: short possession; LP: long; RC: receptions; RV: release velocity; RI: release index.

**TABLE 4:** Descriptive analysis of PL variables by playing positions.

PL variables	FB (4 players)	CD (3 players)	MD (4 players)	WG (4 players)	FW (3 players)
TS (m/s)	7.78 ± 0.42^b^ ^,^ ^c^ ^,^ ^e^	7.20 ± 0.45^a^ ^,^ ^d^	7.01 ± 0.31^a^ ^,^ ^d^ ^,^ ^e^	7.75 ± 0.38^b^ ^,^ ^c^ ^,^ ^e^	7.40 ± 0.40^a^ ^,^ ^c^ ^,^ ^d^
DC (m)	4579.10 ± 228.17^c^ ^,^ ^d^	4436.52 ± 750.83^c^	5509.29 ± 229.52^a^ ^,^ ^b^ ^,^ ^d^ ^,^ ^e^	5235.17 ± 457.54^a^ ^,^ ^c^	4618.90 ± 235.00^c^
WR (m/min)	96.62 ± 14.30^c^ ^,^ ^d^	97.74 ± 6.89^c^	120.83 ± 6.01^a^ ^,^ ^b^ ^,^ ^d^ ^,^ ^e^	113.99 ± 8.47^a^ ^,^ ^c^ ^,^ ^e^	100.56 ± 6.50^c^ ^,^ ^d^
HIDC (m)	1031.65 ± 674.31^c^ ^,^ ^d^	931.86 ± 836.44^c^ ^,^ ^d^	1481.50 ± 1055.23^a^ ^,^ ^b^ ^,^ ^e^	1699.42 ± 1177.15^a^ ^,^ ^b^ ^,^ ^e^	919.20 ± 645.44^c^ ^,^ ^d^
HIDC (m/min)	22.93 ± 14.98^c^ ^,^ ^d^	20.71 ± 18.59^c^ ^,^ ^d^	32.92 ± 23.45^a^ ^,^ ^b^ ^,^ ^e^	37.76 ± 26.16^a^ ^,^ ^b^ ^,^ ^e^	20.43 ± 14.34^c^ ^,^ ^d^
SDC (m)	283.52 ± 248.50^b^ ^,^ ^d^	183.00 ± 226.61^a^ ^,^ ^d^	290.43 ± 422.60^d^	495.42 ± 501.61^a^ ^,^ ^b^ ^,^ ^c^ ^,^ ^e^	189.40 ± 211.62^d^
SDC (m/min)	6.30 ± 5.52^b^ ^,^ ^d^	4.07 ± 5.04^a^ ^,^ ^d^	6.45 ± 9.39^d^	11.01 ± 11.15^a^ ^,^ ^b^ ^,^ ^c^ ^,^ ^e^	4.21 ± 4.70^d^
SP (#)	22.78 ± 8.12^b^ ^,^ ^c^ ^,^ ^d^	11.00 ± 4.53^a^ ^,^ ^c^ ^,^ ^d^ ^,^ ^e^	18.64 ± 6.77^a^ ^,^ ^b^ ^,^ ^d^	29.42 ± 10.36^a^ ^,^ ^b^ ^,^ ^c^ ^,^ ^e^	19.00 ± 6.80^b^ ^,^ ^e^
SP (#/min)	0.50 ± 0.18^b^ ^,^ ^c^	0.23 ± 0.10^a^ ^,^ ^c^ ^,^ ^d^ ^,^ ^e^	0.41 ± 0.14^a^ ^,^ ^b^ ^,^ ^d^	0.64 ± 0.23^b^ ^,^ ^c^ ^,^ ^e^	0.42 ± 0.15^b^ ^,^ ^d^
DTZ1 (m)	2080.17 ± 520.83	2247.76 ± 506.20^c^ ^,^ ^d^	1959.93 ± 377.01^b^ ^,^ ^e^	1977.58 ± 485.29^b^ ^,^ ^e^	2305.50 ± 371.83^c^ ^,^ ^d^
DTZ2 (m)	1326.61 ± 437.59^c^	1400.38 ± 458.67^c^	2067.29 ± 717.29^a^ ^,^ ^b^ ^,^ ^d^ ^,^ ^e^	1556.17 ± 665.86^c^	1394.30 ± 321.25^c^
DTZ3 (m)	448.30 ± 231.43^c^ ^,^ ^d^	478.33 ± 353.57^c^	709.93 ± 208.31^a^ ^,^ ^b^ ^,^ ^e^	684.33 ± 306.94^a^ ^,^ ^e^	460.30 ± 219.13^c^ ^,^ ^d^
DTZ4 (m)	299.83 ± 214.08^c^ ^,^ ^d^	270.52 ± 272.16^c^ ^,^ ^d^	481.14 ± 451.49^a^ ^,^ ^b^ ^,^ ^e^	519.67 ± 393.96^a^ ^,^ ^b^ ^,^ ^e^	269.50 ± 227.99^c^ ^,^ ^d^
DTZ5 (m)	177.91 ± 160.53^d^	141.10 ± 179.67^d^	248.43 ± 361.59	356.67 ± 371.66^a^ ^,^ ^b^ ^,^ ^e^	146.00 ± 171.86^d^
DTZ6 (m)	105.61 ± 94.71^b^ ^,^ ^c^ ^,^ ^e^	41.90 ± 50.24^a^ ^,^ ^d^	42.00 ± 71.40^a^ ^,^ ^d^	138.75 ± 132.37^b^ ^,^ ^c^ ^,^ ^e^	43.40 ± 41.08^a^ ^,^ ^d^
ADA (#)	25.78 ± 8.58	24.05 ± 9.47	29.00 ± 39.00	28.00 ± 28.00	27.90 ± 6.97
ADA (#/min)	0.56 ± 0.18	0.51 ± 0.20	0.66 ± 0.87	0.64 ± 0.64	0.61 ± 0.15

^a^ (significant differences FB).

^b^ (significant differences from CD).

^c^ (significant differences from MD).

^d^ (significant differences from WG).

^e^ (significant differences from FW). TS: top speed; DC: distance covered; WR: work rate; HIDC: high intensity distance covered; DS: distance sprint; SDC: sprint distance covered; SP: number of sprints; DTZ1: Distance Traveled Zone 1; DTZ2: Distance Traveled Zone 2; DTZ3: Distance Traveled Zone 3; DTZ4: Distance Traveled Zone 4; DTZ5: Distance Traveled Zone 5; DTZ6: Distance Traveled Zone 6; ADA: Acceleration/Deceleration Actions.

In the same way, MD also generates the best record for 1T, reaching significant differences with WG (*p* ≤ 0.001) and FW (*p* = 0.01). In contrast, they do not differ significantly with CD (*p* = 0.87) or FB (*p* = 0.80), respectively. In the same way, CD generates the best record for REL without reaching significant differences with FB (*p* = 0.09) or with MD (*p* = 0.34) but obtaining significant differences with FW (*p* ≤ 0.001). CD also generates the best record for TP, with substantial differences with FB (*p* = 0.04), WG (*p* = 0.02) and FW (*p* ≤ 0.001), while no significant differences were found with MD (*p* = 0.12). In the same way, CD generates the best records for SP, LP, RC, RV and RI.

On the other hand, considering PL variables, the highest values ​​by position are found for FB ​​in TS, reaching significant differences with CD (*p* ≤ 0.001), MD (*p* ≤ 0.001) and FW (*p* = 0.03). In contrast, they do not reach significant differences with WG (*p* = 0.92). In the same way, the MD are those that reach the highest records in DC, with significant differences with CD (*p* ≤ 0.001), FB (*p* ≤ 0.001) and WG (*p* = 0.03); WR being the significant differences with CD (*p* ≤ 0.001), FB (*p* ≤ 0.001), WG (*p* = 0.03) and FW (*p* ≤ 0.001); DTZ2 being the significant differences with CD (*p* ≤ 0.001), FB (*p* ≤ 0.001), WG (*p* ≤ 0.001) and FW (*p* ≤ 0.001); DTZ3 being the significant differences with CD (*p* ≤ 0.001), FB (*p* ≤ 0.001) and FW (*p* ≤ 0.001); Similarly, the WG reach the highest records in the following variables: HIDC (finding significant differences with CD (*p* = 0.01), FB (*p* ≤ 0.001) and FW (*p* = 0.02); DS (finding significant differences with CD (*p* = 0.01), FB (*p* = 0.01), MD (*p* = 0.01) and FW (*p* = 0.02)); SP (finding significant differences with CD (*p* = 0.01), FB (*p* = 0.01), MD (*p* = 0.01) and FW (*p* = 0.02)); DTZ4 (finding significant differences with CD (*p* = 0.02), FB (*p* = 0.01) and FW (*p* = 0.03)); DTZ5 (finding significant differences with CD (*p* = 0.01), FB (*p* = 0.01) and FW (*p* = 0.03)); and finally in DTZ6 (finding significant differences with CD (*p* ≤ 0.001), MD (*p* ≤ 0.001) and FW (*p* = 0.01)); and finally, for FW in DTZ1 (finding significant differences with MD (*p* = 0.03) and WG (*p* = 0.04). The number of accelerations (ADA) and accelerations per minute (ADA/min) are the only variables that did not manifest differences by positions.

## Discussion

This study aimed to analyze the activity profile of professional soccer players during friendly matches using TL and PL variables. The main findings include the first-ever description of the TL profile based on players’ positions during matches. Secondly, an intriguing observation emerged from the analysis of TL and PL. Despite significant modifications in the PL profile between the first and second halves of the matches, the TL profile remained remarkably consistent. This suggests that the technical demands placed on players, as reflected by TL variables, are relatively independent of the overall physical load experienced during the match. This finding underscores the complexity of soccer performance, where the interplay between technical and physical elements may not necessarily exhibit a direct correspondence. These results emphasize the independent nature of TL from PL and provide valuable insights into the technical demands of players in different positions during matches. In our study, the ES associated with the significant differences in various PL variables help us better understand the practical significance of these findings. For example, the large effect size (ES: 0.92) for WR (m/min) in the first period highlights a substantial difference in the work rate during this match phase. These effect sizes demonstrate that the observed changes in performance metrics are not only statistically significant but also of practical importance. On the same way, large effect size (ES: 0.57) for DC (m). Additionally, it is worth noting that the effect sizes can provide valuable context when comparing our results to those of previous studies. For instance, the differences in Total Touches and Releases between our study and the work by Yi et al. (2019) may be partially explained by the effect sizes associated with these variables, shedding light on the magnitude of the variations observed.

This is one of the first studies that shed light on providing information on the activity profile based on TL variables of the soccer player in (friendly) matches applying IMU technology. In our study, we have recorded that the soccer player during each part of a friendly match performs an average of 47.0 ± 18.5 and 48.7 ± 26.4 Total Touches and 16.4 ± 9.6 and 15.6 ± 10.7 Releases, respectively. These values ​​are higher for Total Touches and lower for Releases, compared to those obtained by Yi et al. (2019) in a study that analyzed technical performance in the five major European leagues, accounting for the technical actions carried out by outfield players who complete the official match but using semi-automatic cameras. These differences in terms of Total Touches may be due to a greater intensity of the game, with fewer interruptions in the game in higher category matches and fewer necessary touches (Yi et al., 2020), while for Releases, the lower records found in our study may be due to a more excellent combinative game in competitions of a higher competitive level (Yi et al., 2020).

Previous research has shown that players show lower PL records on Match Day-1, contrary to what occurs with TL in the study conducted by Marris et al. (2021). This may be due to an orientation of the training objectives with a more technical-tactical nature (Martín-García et al., 2018; Walker and Hawkins, 2018). Considering these 2 TL variables but focusing on specific positions, the MDs reach the best records for Total Touches. In contrast, the Central Defenders are the ones that reach the highest values for Releases. These values compared to those obtained in the study by Marris et al. (2021), also with IMU technology, are lower, although in said research TL is analyzed within the training microcycle, with Match Day obtaining the highest records for both variables. high, so we found that this specific position was also very prominent in our study. In the same way, analyzing an indicator that combines the volume and intensity of each hit by the player (Release Index), the findings of our study provide absolute values of 22.6 ± 13.0 for the first period and 21.1 ± 14.6 for the second, being lower than those provided by Lewis et al. (2022) in their study with English professional players in training sessions for 25 weeks. The highest values ​​are found in Match Day-4 (128.6 ± 35.7) and Match Day+1 (145 ± 45.2), justifying this great difference with those obtained in our research, firstly, at a distance in time of day of competition and, secondly, to the characteristics of the tasks for Match Day-4, and on the other hand, in Match Day+1, due to the compensation training carried out by the players who have played the fewest minutes and, also, to the characteristics of the training tasks (Arjol-Serrano et al., 2021; Lewis et al., 2022).

Taking this TL indicator (RI) to the analysis by specific positions, the Central Defenders in our study reach the highest values, followed by the Midfielders. These particular positions in the study carried out by Lewis et al. (2022) with IMU technology, but analyzing training tasks by categories, distinguishing: Warm-up; Possessions; Small-Side Game; Tactical Training; Specific Training and Technical Training also find findings that for both positions, the highest values for RI in the Possessions and Tactical Training tasks. Emmonds et al. (2022), in another line of work applying this technology to women’s soccer, also obtain TL values of specific positions during different types of training, distinguishing: Possessions; Intensive Small-Side Games; Extensive Small-Side Games; Tactical Training and Technical Training. These authors show their results without differentiating with the specific positions but find that Total Touches (#), and Total Touches (#/min), reach the highest values during Possession and Tactical Training. In contrast, for Releases (#), Releases (#/min), find them during Tactical Training and Technical Training. The findings in our study and in previous studies mentioned in this article confirm the high degree of specificity reached by training sessions with a high content of technical-tactical components, confirming the need for more research on TL during matches, friendlies and competition to meet the requirements.

Regarding the analysis of the PL, we found in our study results of Work Rate (m/min) similar to those obtained with GPS technology (Torreño, 2017), both at the average level for all positions in the first period (GPS: 113 m/min; IMU: 108.6 m/min), as well as by specific positions Full Back (GPS: 112.8 m/min; IMU: 96.62 m/min), Central Defender (GPS: 103.7 m/min; IMU: 97.74 m/min), Midfielder (GPS: 122.6 m/min; IMU:120.83 m/min), Winger (GPS: 125.6 m/min; IMU: 113.99 m/min) and Fordward (GPS: 119.1 m/min; IMU: 100.56 m/min). The same occurs with Top Speed (m/s), where we obtain that Full Back (7.78 m/s) and Winger (7.75 m/s) are the ones that reach the highest speed peak during a friendly match. In previous studies with GPS technology, it is obtained that Winger (8.6 m/s) is the one that reaches the highest maximum speed during an official match (Suarez-Arrones et al., 2015). Such as in previous studies with other technologies (Carling, 2013; Barnes et al., 2014; Suarez-Arrones et al., 2015; Arjol-Serrano et al., 2021; Teixeira et al., 2021), we found a reduction in High-Intensity Distance Covered/min, and Sprint Distance Covered/min. These results make us assume that the sample with which the TL information has been obtained in this study has a PL behavior very similar to other samples of a higher competitive level, both in friendly and official matches, so it would be interesting to analyze the variables TL in a competitive match to confirm or not the TL profile described in this study and thus obtain a better understanding of the demands of this sport (Marris et al., 2021).

This study has the following limitations: Firstly, the study’s sample size was limited to a single professional team, which may affect the generalizability of the findings to a broader population of soccer players. Including multiple teams from different levels of play would enhance the representativeness and reliability of the results. Secondly, the study focused on friendly matches during the preseason, which may only partially capture the intensity and competitive nature of official matches. The findings might differ in different match contexts, such as league matches or cup competitions. Lastly, it is important to note that this study did not consider other potential factors that could influence TL and PL, such as mental conditions (e.g., stress level, started or not started players, motivation, mental toughness), environmental conditions (e.g., weather, pitch conditions), tactical data (e.g., through passing matrix about the game system, interaction between them) or individual player characteristics (e.g., fitness level, playing style). Future studies could incorporate these variables to provide a more comprehensive understanding of the factors influencing TL and PL in soccer matches.

## Conclusion

The TL profile of professional soccer players, based on their playing position, is found to be independent of the development of PL observed during friendly matches. This profile appears strongly influenced by the game system and the specific role assigned to each position. Therefore, monitoring, quantifying, and controlling TL and PL of soccer players offers a more comprehensive and holistic understanding of the demands in friendly matches compared to solely analyzing PL. Assessing TL during friendly matches enables the differentiation of actions based on players’ positions, which can optimize performance during training sessions.

The practical applications that this entails are.

- Designing training tasks with the TL component depending on the player’s specific position.
- Adapting the volume and intensity of these variables to the needs of the training session within the microcycle, ensuring adequate tapering for the competition day, as is done with PL.
- IMU technology in this context offers a convenient and time-efficient alternative with significant benefits over other existing technologies.

## Data Availability

The raw data supporting the conclusion of this article will be made available by the authors, without undue reservation.
